# Prevalence, incidence, indication, and choice of antidepressants in patients with and without chronic kidney disease: a matched cohort study in UK Clinical Practice Research Datalink

**DOI:** 10.1002/pds.4212

**Published:** 2017-04-11

**Authors:** Masao Iwagami, Laurie A. Tomlinson, Kathryn E. Mansfield, Helen I. McDonald, Liam Smeeth, Dorothea Nitsch

**Affiliations:** ^1^ Department of Non‐Communicable Disease Epidemiology London School of Hygiene and Tropical Medicine London UK

**Keywords:** antidepressants, chronic kidney disease, prevalence, incidence, depression

## Abstract

**Purpose:**

People with chronic kidney disease (CKD) have an increased prevalence of depression, anxiety, and neuropathic pain. We examined prevalence, incidence, indication for, and choice of antidepressants among patients with and without CKD.

**Methods:**

Using the UK Clinical Practice Research Datalink, we identified patients with CKD (two measurements of estimated glomerular filtration rate < 60 mL/min/1.73m^2^ for ≥3 months) between April 2004 and March 2014. We compared those with CKD to a general population cohort without CKD (matched on age, sex, general practice, and calendar time [index date]). We identified any antidepressant prescribing in the six months prior to index date (prevalence), the first prescription after index date among non‐prevalent users (incidence), and recorded diagnoses (indication). We compared antidepressant choice between patients with and without CKD among patients with a diagnosis of depression.

**Results:**

There were 242 349 matched patients (median age 76 [interquartile range 70–82], male 39.3%) with and without CKD. Prevalence of antidepressant prescribing was 16.3 and 11.9%, and incidence was 57.2 and 42.4/1000 person‐years, in patients with and without CKD, respectively. After adjusting for confounders, CKD remained associated with higher prevalence and incidence of antidepressant prescription. Regardless of CKD status, selective serotonin reuptake inhibitors were predominantly prescribed for depression or anxiety, while tricyclic antidepressants were prescribed for neuropathic pain or other reasons. Antidepressant choice was similar in depressed patients with and without CKD.

**Conclusions:**

The rate of antidepressant prescribing was nearly one and a half times higher among people with CKD than in the general population. © 2017 The Authors. Pharmacoepidemiology & Drug Safety Published by John Wiley & Sons Ltd

## Introduction

Antidepressants are among the most commonly prescribed classes of medication in industrialized countries, including the USA^1^ and UK.^2^ The recent increase in the prescription of antidepressants is dramatic, with an average 10% increase per year from 1998 to 2010.^3^ Antidepressants can be prescribed not only for depressive symptoms but also for other conditions such as anxiety and neuropathic pain.^4^ In addition, off‐label use of antidepressants is common for chronic pain, including non‐neuropathic pain, and conditions where non‐specific sedation is required.^5^, ^6^, ^7^

Chronic kidney disease (CKD), an impairment of kidney structure or function, is now recognized as a major public health problem.^8^ Chronic kidney disease is associated with a range of comorbidities including obesity, hypertension, diabetes, and cardiovascular disease.^9^, ^10^ Level of kidney function, expressed as estimated glomerular filtration rate (eGFR), is closely associated with increased risk of death, cardiovascular events, and hospitalization.^11^

Chronic kidney disease is also associated with a range of mental health problems including anxiety^12^ and depression;^13^ almost one quarter of adults with pre‐dialysis CKD are depressed. These conditions may be due to co‐existing chronic diseases such as diabetes and heart failure, which are also associated with depression and anxiety symptoms,^14^, ^15^ or directly related to CKD. In addition, other indications for antidepressants such as chronic pain and insomnia are more common among patients with CKD.^16^, ^17^

Patients with CKD are frequently excluded from clinical trials,^18^, ^19^ and concerns have been recently raised about the lack of knowledge regarding how kidney function is related to adverse effects of antidepressants.^20^, ^21^ Despite this, there has been no systematic research investigating frequency and patterns of antidepressant prescribing among patients with CKD. Understanding how antidepressants are actually prescribed in patients with CKD, compared to those without CKD, is important groundwork for the planning of future studies on the safety of antidepressants in this population. Therefore, we aimed to compare the frequency (prevalence and incidence), indications for, and choice of antidepressant prescription between patients with and without CKD, in the UK general population.

## Methods

### Data sources

The Clinical Practice Research Datalink (CPRD) is a database of routinely recorded primary care electronic health record data from 7% of the UK population.^22^ The database includes the following data: patient demographics; diagnoses; prescriptions; laboratory test results; and referrals made by general practitioners (GPs). Diseases can be identified using diagnostic codes (Read codes) recorded in routine data. We used CPRD linked to additional data sources: the inpatient Hospital Episodes Statistics (HES) database to provide data on ethnicity (to improve data completeness);^23^ Office for National Statistics (ONS) data for mortality; and Index of Multiple Deprivation (IMD) data for deprivation indices. We obtained study approval from the institutional review board of the London School of Hygiene and Tropical Medicine (reference: 9196), as well as the Independent Scientific Advisory Committee, which oversees research involving CPRD data (Protocol 15_219R). Informed consent from individual patients was waived because the data are anonymous.

### Study population and matched cohort

All adults (age 18 or older) alive and contributing to HES‐linked CPRD anytime from 1 April 2004 to 31 March 2014 were potentially eligible for inclusion. Patients were eligible for inclusion at the latest of: one year after practice registration,^24^ the date that the practice reached CPRD quality control standards, or 1 April 2004. Patients were no longer eligible for follow‐up at the first of renal replacement therapy (RRT) initiation, death, change of practice, last data collection from the practice, or 31 March 2014. We excluded patients already receiving RRT (hemodialysis, peritoneal dialysis, and kidney transplantation) prior to cohort entry.

First, we identified patients with CKD based on two consecutive measurements of eGFR <60 mL/min/1.73 m^2^ more than three months apart.^25^ Estimated GFR was calculated from serum creatinine values recorded in CPRD, using the Chronic Kidney Disease Epidemiology Collaboration equation.^26^ Patients, including those who had CKD before April 2004, were included in the cohort on the date when they first satisfied the CKD definition (i.e. second eGFR <60 mL/min/1.73 m^2^) during eligible follow‐up (index date).

Next, as a control group, we selected at random patients without CKD from the general population. Because (i) CKD status largely depends on age and sex, and (ii) pattern of antidepressant prescription is expected to depend on general practice and calendar time, we matched controls to patients with CKD by age (same year of birth), sex, general practice, and calendar time. Each control entered the cohort on the same index date as their CKD counterpart. Individuals selected as controls (i.e. non‐CKD patients) may be found to have CKD later; in this situation, they were censored as a control at the time of satisfying CKD definition and contributed separately as an incident patient with CKD from that time point forward (with their own matched control).

### Prevalence and incidence of antidepressant prescription

We estimated the prevalence of existing users of antidepressants, defined as receiving an antidepressant prescription within six months prior to the index date. Incidence of antidepressant prescription was based on the first antidepressant prescription after index date, after exclusion of existing users.^27^

### Covariates

In order to examine the independent association between CKD status and antidepressant prescription, we considered baseline characteristics of patients: age and sex; ethnicity; socio‐economic status (SES); smoking status; body mass index (BMI); and common chronic physical illnesses that are considered to be associated with mental health conditions (diabetes mellitus, congestive heart failure, myocardial infarction, stroke, chronic obstructive pulmonary disease, rheumatoid arthritis, cancer, Parkinson's disease, and epilepsy).^28^, ^29^ Based on previous studies using UK primary care data,^30^, ^31^ we classified patients with no record of ethnicity as white. Socio‐economic status was assigned by quintile at an individual level, using 2010 ONS estimates of the IMD (a composite area‐level marker of deprivation).^32^ For patients with missing individual‐level SES, we used the SES for the patient's general practice. Smoking status and BMI were assigned using the data recorded closest to the index date. We defined each chronic physical illness as present if a relevant diagnosis code of that illness was recorded at least once before a patient's index date.

### Indication

We identified morbidity codes for three common diagnoses suggesting indications for antidepressant prescription:^4^ depression, anxiety, and neuropathic pain (included in Appendix 1). We included symptom codes as well as diagnostic codes because GPs in the UK commonly use symptom codes (e.g. “depressive symptoms”, “anxiousness”) rather than definitive diagnostic codes (e.g. “major depression”, “general anxiety disorder”).^33^, ^34^, ^35^ We included codes recorded by GPs any time prior to the first antidepressant prescription until three months later, to account for possible time lag in recording diagnosis codes in electronic health records.^36^, ^37^ We categorized type of antidepressant, according to British National Formulary headings, into the following categories:^4^ selective serotonin reuptake inhibitors (SSRIs), tricyclic antidepressants (TCAs), or other antidepressants. Monoamine oxidase inhibitors were grouped into other antidepressants because of a small number of prescriptions. For each type of antidepressant, we identified the proportion of patients with each indication as well as those with none of the three indications.

### Choice of antidepressants and initial prescription dose

There are 26 antidepressants currently available in the UK, only a few are indicated for anxiety and neuropathic pain, whilst all 26 are indicated for depressive conditions.^4^ Therefore, we restricted this analysis to patients with a recorded diagnosis of depression. We compared the pattern of antidepressant choice (the proportion of patients prescribed each antidepressant as their first incident prescription) between depressed patients with and without CKD. We also compared the initial dose prescribed in those with and without CKD to examine whether antidepressants were started at a lower dose in patients with CKD than those without.

### Statistical analyses

We compared the baseline characteristics of patients with and without CKD using χ^2^ tests. We calculated crude prevalence and incidence rates for antidepressant prescribing. We then conducted a conditional logistic regression analysis (to account for matching) to investigate the association between CKD status and *prevalence* of antidepressant prescription. After excluding existing users of antidepressants (meaning matching was no longer maintained), we conducted an unconditional Poisson regression analysis to investigate the association between CKD status and *incidence* of new antidepressant prescription, adjusting for age, sex, and financial year, and taking account of clustering by general practice using robust standard errors. We adjusted for financial year (by including financial year as a categorical variable, i.e. from 1 April to 31 March for each year) because the frequency of antidepressant prescribing has been increasing year by year.^3^ We further adjusted for ethnicity, SES, smoking status, and BMI, and, then, in a fully adjusted model, also included chronic physical illnesses. In models including smoking status and BMI, we included an additional absent category for those with no recorded smoking status or BMI. In a subsequent sensitivity analysis, we dropped all those with missing smoking or BMI status. All the data management and statistical analyses were conducted using STATA version 14 (Stata Corp, Texas).

### Renal function subgroup analyses

To examine the association between severity of kidney function and antidepressant prescribing, we classified patients with CKD according to the level of kidney function on the index date into two categories: eGFR 30–59 (CKD stage 3), and <30 mL/min/1.73 m^2^ (CKD stage 4 and 5).^25^ In patients without CKD, we differentiated patients with and without serum creatinine results recorded in CPRD prior to index date, because these subgroups are expected to be systematically different due to testing incentives for those at risk of CKD in the UK Quality and Outcomes Framework.^38^ To compare the prevalence of existing users of antidepressants between subgroups of renal function, we used an unconditional logistic regression analysis, adjusting for age, sex, and financial year, and taking account of clustering by general practice using robust standard errors. We also repeated all other principal analyses (described under ‘Statistical analyses’ subheading) using renal function subgroups.

### Additional analyses

Any difference in the duration of follow‐up lengths between patients with and without CKD may affect the likelihood of starting antidepressants. Therefore, as a post hoc analysis, we compared the proportion of patients starting antidepressants within the first six months of follow‐up in those with and without CKD.

We undertook a further analysis to investigate whether patients with CKD were more likely to start antidepressants for the first episode of depression in their life, or for a recurrent episode of depression. In CPRD, GPs routinely record a patient's past medical history shortly after registration with a new practice, and, therefore, a previous episode of depression would be recorded between CPRD registration and index date of the study (as index dates need to be at least one year after CPRD registration by our definition). Therefore, in patients starting antidepressants with a recorded diagnosis of depression, we compared the proportion of those with and without CKD who had: (i) their first depression diagnosis in CPRD recorded between CPRD registration and index date (more likely to suggest a recurrence); and (ii) their first depression diagnosis recorded in CPRD after index date (more likely to suggest the first ever depression diagnosis).

## Results

**Figure 1:**
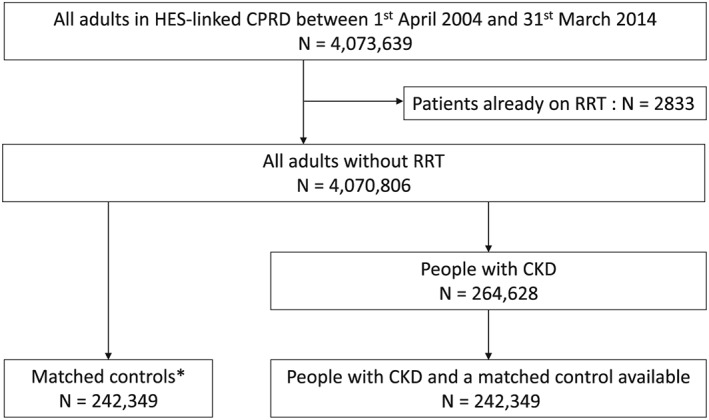
Flow chart for the selection of matched patients with and without chronic kidney disease. CKD = chronic kidney disease, CPRD = clinical practice research datalink, HES = hospital episode statistics, RRT = renal replacement therapy. *Matched cohort: randomly selected individuals without chronic kidney disease matched for age, sex, general practice, and calendar time

Among 4 070 806 eligible patients (median age 39 [IQR 27–56], male 48.8%), we identified 264 628 patients with CKD (median age 77 [IQR 71–83], male 38.7%) (Figure 1). Of those with CKD, 242 349 (91.6%) (median age 76 [IQR 70–82], male 39.3%) were matched with a control patient without CKD who had the same age, sex, and general practice on the index date of their CKD counterpart. Unmatched patients with CKD (*n* = 22 279) were older and more likely to be female (median age 88 [IQR 84–92], male 31.5%). Of the 242 349 matched control patients without CKD, 41 151 (17.0%) were subsequently found to have CKD.

Compared to patients without CKD, patients with CKD were more likely to be deprived, ex‐smokers, and overweight (BMI ≥25 kg/m^2^) (Table 1). Chronic physical illnesses, except for Parkinson's disease and epilepsy, were more common among patients with CKD.

**Table 1:** Baseline characteristics of matched patients with and without chronic kidney disease

	Patients without CKD *N* = 242 349	Patients with CKD *N* = 242 349	*p* Value
	*n* (%)	*n* (%)	
Age (years):			1.000
<55	6845 (2.8)	6845 (2.8)	
55–64	23 556 (9.7)	23 556 (9.7)	
65–74	71 112 (29.3)	71 112 (29.3)	
75–84	102 594 (42.3)	102 594 (42.3)	
≥85	38 242 (15.8)	38 242 (15.8)	
Sex (male):	95 318 (39.3)	95 318 (39.3)	1.000
Ethnicity:			
White/not‐recorded*	238 533 (98.4)	238 138 (98.3)	<0.001
South Asian	1796 (0.7)	2317 (1.0)	
Black	1156 (0.5)	1060 (0.4)	
Other ethnicity	864 (0.4)	834 (0.3)	
Socio‐economic status**:			
1 (least deprived)	56 800 (23.4)	53 034 (21.9)	<0.001
2	61 647 (25.4)	60 501 (25.0)	
3	50 466 (20.8)	50 709 (20.9)	
4	42 221 (17.4)	44 692 (18.4)	
5 (most deprived)	31 215 (12.9)	33 413 (13.8)	
Smoking status:			<0.001
Non‐smoker	92 363 (38.1)	80 721 (33.3)	
Ex‐smoker	107 737 (44.5)	131 510 (54.3)	
Current‐smoker	36 338 (15.0)	29 243 (12.1)	
Missing	5911 (2.4)	875 (0.4)	
Body mass index (kg/m^2^):			<0.001
<18.5	6638 (2.7)	4562 (1.9)	
18.5–25	85 473 (35.3)	70 102 (28.9)	
≥25	80 458 (33.2)	88 083 (36.4)	
≥30	40 326 (16.6)	63 183 (26.1)	
Missing	29 454 (12.2)	16 419 (6.8)	
Chronic physical illnesses:			
Diabetes mellitus	24 292 (10.0)	52 802 (21.8)	<0.001
Congestive heart failure	7581 (3.1)	23 774 (9.8)	<0.001
Myocardial infarction	11 459 (4.7)	25 746 (10.6)	<0.001
Stroke	12 243 (5.1)	19 982 (8.3)	<0.001
Chronic obstructive pulmonary disease	14 996 (6.2)	18 229 (7.5)	<0.001
Rheumatoid arthritis	4270 (1.8)	6031 (2.5)	<0.001
Cancer	47 431 (19.6)	54 450 (22.5)	<0.001
Parkinson's disease	2691 (1.1)	2293 (1.0)	<0.001
Epilepsy	3972 (1.6)	3682 (1.5)	0.001

CKD = chronic kidney disease.

* White/not‐recorded: 136 119 (56.2%) and 140 784 (58.1%) patients with and without CKD, respectively, had no recorded ethnicity.

** Socio‐economic status: 259 (0.1%) and 272 (0.1%) patients with and without CKD, respectively, did not have individual‐level data; we therefore used the socio‐economic status of their general practice.

Prevalence of existing use of antidepressants at index date was 16.3 and 11.9% in patients with and without CKD, respectively (Table 2). The incidence rate of new antidepressant prescription was 57.2 and 42.4/1000 person‐years in patients with and without CKD, respectively (Table 3). After adjusting for confounding, CKD remained positively associated with increased prevalence and incidence of antidepressant prescribing (Tables 2 and 3). Our results were similar to those in the main analysis after excluding patients with missing smoking status and BMI (data not shown).

**Table 2:** Prevalence of antidepressant prescription by chronic kidney disease status

	No. of patients receiving antidepressants in the past 6 months	Prevalence % (95%CI)	Adjusted odds ratio (95%CI)		
			Model 1*	Model 2**	Model 3***
Patients without CKD (*N* = 242 349)	28 738	11.9 (11.7–12.0)	1 (Reference)	1 (Reference)	1 (Reference)
Patients with CKD (*N* = 242 349)	39 428	16.3 (16.1–16.4)	1.46 (1.43–1.48)	1.43 (1.41–1.46)	1.35 (1.32–1.37)

CI = confidence interval, CKD = chronic kidney disease.

* Model 1: Accounted for the matched nature of the groups (age, sex, general practice, and calendar time) in conditional logistic regression analysis.

** Model 2: Model 1 + adjusted by ethnicity, socio‐economic status, smoking status, and body mass index.

*** Model 3: Model 2 + adjusted by chronic physical illnesses.

**Table 3:** Incidence of new antidepressant prescription by chronic kidney disease status

	Total follow‐up length (person‐years)	No. of patients starting antidepressants	Incidence rate (/1000 person‐years) (95%CI)	Adjusted rate ratio (95%CI)		
				Model 1*	Model 2**	Model 3***
Patients without CKD (*N* = 213 611)	774 660	32 846	42.4 (41.9–42.9)	1 (Reference)	1 (Reference)	1 (Reference)
Patients with CKD (*N* = 202 921)	794 150	45 394	57.2 (56.6–57.7)	1.35 (1.33–1.37)	1.30 (1.28–1.32)	1.25 (1.23–1.26)

CI = confidence interval, CKD = chronic kidney disease, IQR = interquartile range.

* Model 1: Adjusted by age, sex, and financial year, and taking account of clustering by general practice with robust standard errors using unconditional Poisson regression analysis.

** Model 2: Model 1 + adjusted by ethnicity, socio‐economic status, smoking status, and body mass index.

*** Model 3: Model 2 + adjusted by chronic physical illnesses.

The pattern of recorded diagnoses was broadly similar between patients with and without CKD (Table 4). Regardless of CKD status, the majority of patients prescribed SSRIs had recorded diagnoses of depression or anxiety, while TCAs were prescribed for neuropathic pain or other reasons. Among patients with a recorded diagnosis of depression, the choice of antidepressant was similar between patients with and without CKD (Table 5). Irrespective of CKD status, the most commonly prescribed antidepressant was citalopram, followed by amitriptyline, fluoxetine, sertraline, and mirtazapine. There was no clear evidence that antidepressants were started at a reduced dose in patients with CKD, compared to those without CKD.

**Table 4:** Recorded diagnoses for patients prescribed antidepressants stratified by chronic kidney disease status and type of antidepressant

	Patients without CKD (*N* = 32 846)			Patients with CKD (*N* = 45 394)		
	SSRI *N* = 12 924	TCA *N* = 17 672	Others *N* = 2250	SSRI *N* = 17 992	TCA *N* = 24 262	Others *N* = 3140
Depression, *n* (%)*	8123 (62.9)	4430 (25.1)	1035 (46.0)	11 363 (63.2)	6257 (25.8)	1456 (46.4)
Anxiety, *n* (%)*	4843 (37.5)	3902 (22.1)	708 (31.5)	6131 (34.1)	5055 (20.8)	935 (29.8)
Neuropathic pain, *n* (%)*	625 (4.8)	2536 (14.4)	152 (6.8)	997 (5.5)	3491 (14.4)	209 (6.7)
None of the above, *n* (%)	3188 (24.7)	9699 (54.9)	894 (39.7)	4683 (26.0)	13 259 (54.7)	1256 (40.0)

CKD = chronic kidney disease, SSRI = selective serotonin reuptake inhibitor, TCA = tricyclic antidepressants.

* Percentages are column percentages. Each patient may have one or more recorded diagnosis.

**Table 5:** Choice of antidepressants and initial prescription dose for patients with diagnosed depression by chronic kidney disease status

	Patients without CKD *N* = 13 588		Patients with CKD *N* = 19 076	
	*n* (%)*	Median initial dose (mg/day) [IQR]	*n* (%)*	Median initial dose (mg/day) [IQR]
Selective serotonin reuptake inhibitors				
Citalopram	4934 (36.3)	10 [10–20]	7070 (37.1)	10 [10–20]
Escitalopram	353 (2.6)	5 [5–10]	429 (2.3)	5 [5–10]
Fluoxetine	1651 (12.2)	20 [20–20]	2270 (11.9)	20 [20–20]
Fluvoxamine	<5 (<0.1)	n/a	<5 (<0.1)	n/a
Paroxetine	132 (1.0)	20 [20–20]	144 (0.8)	20 [20–20]
Sertraline	1053 (7.8)	50 [50–50]	1449 (7.6)	50 [50–50]
Tricyclic and related antidepressants				
Amitriptyline	3506 (25.8)	10 [10–20]	5024 (26.3)	10 [10–15]
Clomipramine	26 (0.2)	25 [10–37.5]	27 (0.1)	20 [10–37.5]
Dosulepin	407 (3.0)	37.5 [25–75]	512 (2.7)	37.5 [25–75]
Doxepin	19 (0.1)	25 [25–37.5]	24 (0.1)	25 [20–30]
Imipramine	30 (0.2)	25 [10–30]	45 (0.2)	25 [10–30]
Lofepramine	113 (0.8)	70 [70–140]	186 (1.0)	70 [70–140]
Nortriptyline	94 (0.7)	15 [10–15]	158 (0.8)	10 [10–15]
Trimipramine	15 (0.1)	25 [10–37.5]	26 (0.1)	25 [20–50]
Mianserin	7 (0.1)	30 [30–30]	5 (<0.1)	30 [30–30]
Trazodone	213 (1.6)	50 [50–100]	250 (1.3)	50 [50–75]
Monoamine oxidase inhibitors**	<5 (<0.1)	n/a	<5 (<0.1)	n/a
Other antidepressants:				
Agomelatine	<5 (<0.1)	n/a	<5 (<0.1)	n/a
Duloxetine	98 (0.7)	40 [30–60]	169 (0.9)	40 [30–60]
Flupentixol	63 (0.5)	1 [0.5–1]	88 (0.5)	1 [0.5–1]
Mirtazapine	758 (5.6)	15 [15–15]	1045 (5.5)	15 [15–15]
Reboxetine	<5 (<0.1)	n/a	<5 (<0.1)	n/a
Venlafaxine	85 (0.6)	75 [75–75]	97 (0.5)	75 [75–75]
Two or more different antidepressants	27 (0.2)	n/a	53 (0.3)	n/a

CKD = chronic kidney disease, IQR = interquartile range.

* Cell counts less than five have been suppressed to preserve patient privacy.

** Phenelzine, isocarboxazid, tranylcypromine, and moclobemide are combined due to small sample sizes.

When we repeated our analyses in subgroups of renal function (Appendix 2 Tables 1–5), as the level of kidney function decreased, patients tended to be older and sicker. Among patients without CKD, those with serum creatinine results recorded in CPRD were sicker than those without. Prevalence and incidence of antidepressant prescribing increased among people with more severe kidney function: prevalence was 16.1 and 18.3%, and incidence was 56.9 and 62.3/1000 person‐years in patients with eGFR 30–59 and <30 mL/min/1.73 m^2^, respectively. This trend remained after adjusting for confounders. Patterns of indication for and choice of antidepressant, as well as initial prescription dose, were broadly similar for patients with different levels of kidney function.

In additional analyses with follow‐up restricted to the first six months, the percentage of patients starting antidepressants was higher amongst patients with CKD (3.5%; 7155/202 921) than amongst those without it (2.5%; 5233/213 611) (*p* < 0.001).

The proportion of patients starting antidepressants with their first depression diagnosis recorded between CPRD registration and index date was larger among patients with CKD (5.8%; 11 781/202 921) than those without CKD (4.0%; 8476/213 611) (*p* < 0.001). Similarly, the proportion of patients starting antidepressants with their first depression diagnosis recorded in CPRD after index date was larger among patients with CKD (3.6%; 7295/202 921) than those without CKD (2.4%; 5112/213 611) (*p* < 0.001). These results suggest that patients with CKD are more likely than those without CKD to start antidepressants both for recurrent episodes of depression, and for their first ever episode of depression.

## Discussion

### Main findings

In this large study, we found that patients with CKD were more likely than patients without CKD to be receiving an antidepressant, or among non‐users, to start one during follow‐up. The increase in prevalence and incidence was graded according to severity of kidney function, and the association remained after adjusting for baseline characteristics including chronic physical illnesses. The pattern of indication for and choice of antidepressants, as well as initial prescription dose, were broadly similar between patients with and without CKD.

### Strengths and limitations

We used a detailed source of routinely collected data that is representative of UK population demographics.^22^ In the UK, GPs manage the vast majority of non‐refractory cases of mental health disorders,^39^, ^40^ and even when patients see psychiatrists in secondary care, prescriptions are usually administered by primary care.^41^ Therefore, we expect that most antidepressant prescriptions are captured in CPRD. To better understand the characteristics of patients with CKD, we used a comparison group of patients without CKD matched on age, sex, general practice, and calendar time. Although previous studies suggested that the proportion of patients with CKD receiving antidepressants may be high as an absolute value,^42^, ^43^ we are not aware of any study that has directly compared frequency and patterns of antidepressant prescribing between patients with and without CKD. We defined CKD using eGFR calculated from serum creatinine measurement. This method is more accurate than using recorded diagnosis of CKD, which has low sensitivity for detecting people with CKD in UK primary care databases.^44^

We must acknowledge several limitations of our study. First, serum creatinine testing in primary care is not universal—currently, it is only recommended and incentivized for people who are considered to be at risk for CKD.^9^, ^38^ We may have misclassified patients with unmeasured CKD to the matched control cohort, which could dilute the true association between CKD and antidepressant prescription. However, a recent study showed that the prevalence of CKD identified in CPRD is similar to that estimated in a nationally representative survey (Health Survey for England), suggesting that most CKD patients are captured in CPRD.^45^ Second, although we adjusted for important confounders that may be associated with mental health conditions,^28^, ^29^ the observed association between CKD status and the prevalence/incidence of antidepressant prescribing could be influenced by residual confounding due to un‐coded poor health status or access to talking therapies. Third, we examined three common diagnoses associated with antidepressant use (depression, anxiety, and neuropathic pain). However, for patients with two or more different diagnoses (e.g. depression and neuropathic pain), it was not possible to determine the most likely indication for antidepressant prescription because diagnosis and prescription records are separate in CPRD. Also, patients may have received antidepressants for other reasons, such as non‐neuropathic pain and insomnia, but reliable identification of these conditions has not been established in CPRD. Finally, we demonstrated that the initial dose of antidepressant prescribed was similar in depressed patients with and without CKD. However, this does not ensure that the subsequent dose was also similar between those with and without CKD (as doctors may increase or decrease antidepressant dose after initial prescription, according to perceived effectiveness or side effects).

### Comparison with other studies

Two studies conducted in the USA have examined antidepressant use in patients with CKD.^42^, ^43^ The Chronic Renal Insufficiency Cohort study investigated the proportion of patients with CKD receiving an antidepressant at recruitment.^43^ Of 3853 participants, 700 (18.2%) were taking antidepressants. This number is close to the prevalence of existing users of antidepressants in patients with CKD (16.3%) found in our study. Another US cohort study showed that around 30% of patients with CKD (with or without diagnosis of depression) were receiving antidepressants at any time during a 2‐year period between 2004 and 2006.^42^ These antidepressant users appeared to include both existing and new users of antidepressants. Our study demonstrated the incident rate of antidepressant prescription at 57.2/1000 person‐years in patient with CKD. Together with the prevalence of existing users (16.3%), the cumulative effect of this was consistent with over 30% of CKD patients exposed to antidepressants during follow‐up. Neither US study included a comparison group of patients without CKD in order to compare prescribing in CKD patients to that in the general population. Indication and choice of antidepressants were also not examined.

### Explanation of findings and implication for future studies

Patients with mild CKD generally do not have related physical symptoms. However, a previous study has suggested that negative perception of CKD is associated with depression and lower quality of life, even in the early stages of CKD.^46^ Patients with more advanced CKD (eGFR <30 mL/min/1.73m^2^) tend to have symptoms including fatigue, nausea, sleep disturbances, itching, and peripheral neuropathy, any of which could influence quality of life and mental health. This is in line with our finding that patients with advanced CKD were more likely to be prescribed antidepressants, even without a specifically coded diagnosis of depression and anxiety.

While most SSRIs were associated with a coded diagnosis of depression or anxiety, more than half of patients starting TCAs (mostly amitriptyline) did not have any recorded diagnoses of depression, anxiety, or neuropathic pain. Amitriptyline may have been predominantly prescribed as an off‐label indication for non‐psychiatric conditions such as chronic pain and insomnia.^5^, ^6^, ^7^ When restricted to patients with a coded diagnosis of depression, SSRIs accounted for the majority of antidepressant prescribing, which is in keeping with current guidelines for management of depression.^39^ Patterns of antidepressant choice did not differ substantially according to CKD status or level of kidney function. This is probably because to date there is no evidence of greater efficacy or safety concerns for specific antidepressants among patients with CKD.^20^, ^21^

Increased adverse events as renal function declines are an important concern. For example, amitriptyline clearance is reduced in patients with decreased kidney function.^47^ As a result, amitriptyline may accumulate, causing serious adverse outcomes through neurotoxicity^48^ and cardiotoxicity.^49^ Another example is the potential amplification of bleeding risk both with use of SSRIs and with decreased kidney function itself.^50^ Finally, the results of our analyses stratified by severity of renal function demonstrate that many patients are prescribed antidepressants at levels of renal function below those where cessation is recommended by manufacturers (e.g. eGFR <30 mL/min/1.73 m^2^). According to the British National Formulary,^4^ escitalopram, paroxetine, sertraline, imipramine, lofepramine, trazodone, duloxetine, mirtazapine, and venlafaxine should be used with caution or avoided in those with reduced renal function, but our real‐world data suggest that these drugs are prescribed similarly in patients with moderately or severely decreased kidney function, compared to those with normal kidney function. Better evidence regarding the potential adverse effects of these drugs for patients with decreased kidney function is needed.

## Conclusions

This study using a large UK database suggests that patients with CKD are more likely to be prescribed antidepressants than the general population, whilst prescribing patterns did not appear to be influenced by kidney function. These real‐world data emphasize the need for research investigating the potential adverse effects of antidepressant therapy in people with decreased kidney function.

## Conflict of Interest

The authors declare no conflict of interest.

**Key Points**

- This study examined details of antidepressant prescribing in patients with chronic kidney disease using a large, contemporary UK database of routine medical record data. We defined chronic kidney disease using serum creatinine measurements and compared people with and without chronic kidney disease matched for age, sex, general practice, and calendar time.
- Patients with chronic kidney disease were exposed to antidepressants more frequently; with higher prevalence and incidence of antidepressant prescribing than the general population. The positive association between chronic kidney disease and increased frequency of antidepressant prescribing remained after adjusting for measured confounders such as diabetes and cardiovascular disease.
- Among patients starting antidepressants, indication for antidepressant prescription (recorded diagnoses of depression, anxiety, or neuropathic pain) was similar between patients with and without chronic kidney disease. Antidepressant choice was also similar between depressed patients with and without chronic kidney disease.

## Author Contributions

M.I. planned the study, carried out the data extraction, processing and analysis, and drafted the manuscript. D.N. and L.T. contributed substantially to the study design, interpretation of the results, and writing of the manuscript. K.M. and H.M. supported the data processing and writing of the manuscript. L.S. was involved in discussions of the analytical approach to this study and made comments on the results. All authors read and approved the final manuscript.

## Supporting information


**Appendix S1.** List of diagnosis codes indicative of depression, anxiety, and neuropathic pain in Clinical Practice Research Datalink.

**Appendix S2.** Subgroup analyses according to level of kidney function (among patients with CKD) and creatinine measurement (among patients without CKD).
