# Supplementary material for: Prevalence, incidence, indication, and choice of antidepressants in patients with and without chronic kidney disease: a matched cohort study in UK Clinical Practice Research Datalink
Source: Pharmacoepidemiol Drug Saf. 2017 Apr 11;26(7):792–801. doi: 10.1002/pds.4212 (PMC5516188; doi:10.1002/pds.4212)
Supplement: Supplementary file 1 — Appendix S1. List of diagnosis codes indicative of depression, anxiety, and neuropathic pain in Clinical Practice Research Datalink. [file PDS-26-792-s001.docx]

**Appendix 1.** List of diagnosis codes indicative of depression, anxiety, and neuropathic pain in Clinical Practice Research Datalink.

| Read code | Medcode* | Read term |
| --- | --- | --- |
| Depression: | | |
| E2B..00 | 324 | Depressive disorder NEC |
| Eu32z11 | 543 | [X]Depression NOS |
| E112.14 | 595 | Endogenous depression |
| E200300 | 655 | Anxiety with depression |
| E135.00 | 1055 | Agitated depression |
| E204.00 | 1131 | Neurotic depression reactive type |
| Eu31.11 | 1531 | [X]Manic-depressive illness |
| E290.00 | 1533 | Brief depressive reaction |
| 2257.00 | 1908 | O/E - depressed |
| 1B17.00 | 1996 | Depressed |
| 1B1N.00 | 2147 | Poor self esteem |
| E11..12 | 2560 | Depressive psychoses |
| E204.11 | 2639 | Postnatal depression |
| 1465.00 | 2716 | H/O: depression |
| 62T1.00 | 2923 | Puerperal depression |
| 1B17.12 | 2930 | C/O - feeling unhappy |
| Eu32z00 | 2970 | [X]Depressive episode, unspecified |
| E2B0.00 | 2972 | Postviral depression |
| Eu32z12 | 3291 | [X]Depressive disorder NOS |
| Eu33.00 | 3292 | [X]Recurrent depressive disorder |
| E2B1.00 | 4323 | Chronic depression |
| Eu32.00 | 4639 | [X]Depressive episode |
| E115.00 | 4677 | Bipolar affective disorder, currently depressed |
| Eu31500 | 4732 | [X]Bipolar affect dis cur epi severe depres with psyc symp |
| 1B17.11 | 4824 | C/O - feeling depressed |
| Eu53012 | 4979 | [X]Postpartum depression NOS |
| E112.11 | 5879 | Agitated depression |
| Eu32z14 | 5987 | [X] Reactive depression NOS |
| E113700 | 6482 | Recurrent depression |
| E112.12 | 6546 | Endogenous depression first episode |
| Eu32y00 | 6854 | [X]Other depressive episodes |
| E113.11 | 6932 | Endogenous depression - recurrent |
| E112.13 | 6950 | Endogenous depression first episode |
| E112z00 | 7011 | Single major depressive episode NOS |
| 1BJ..00 | 7412 | Loss of confidence |
| Eu32.13 | 7604 | [X]Single episode of reactive depression |
| Eu34113 | 7737 | [X]Neurotic depression |
| Eu41211 | 7749 | [X]Mild anxiety depression |
| Eu34100 | 7953 | [X]Dysthymia |
| E130.00 | 8478 | Reactive depressive psychosis |
| Eu34111 | 8584 | [X]Depressive neurosis |
| Eu33.15 | 8826 | [X]SAD - Seasonal affective disorder |
| Eu33.11 | 8851 | [X]Recurrent episodes of depressive reaction |
| Eu33.13 | 8902 | [X]Recurrent episodes of reactive depression |
| 1BT..11 | 8928 | Low mood |
| Eu32.11 | 9055 | [X]Single episode of depressive reaction |
| E11z200 | 9183 | Masked depression |
| Eu32100 | 9211 | [X]Moderate depressive episode |
| Eu32200 | 9667 | [X]Severe depressive episode without psychotic symptoms |
| 1B1U.00 | 9796 | Symptoms of depression |
| 1BT..00 | 10015 | Depressed mood |
| Eu34112 | 10290 | [X]Depressive personality disorder |
| 1B1U.11 | 10438 | Depressive symptoms |
| E211200 | 10455 | Depressive personality disorder |
| E112.00 | 10610 | Single major depressive episode |
| Eu32400 | 10667 | [X]Mild depression |
| Eu32y11 | 10720 | [X]Atypical depression |
| E118.00 | 10825 | Seasonal affective disorder |
| Eu33212 | 11252 | [X]Major depression, recurrent without psychotic symptoms |
| Eu33211 | 11329 | [X]Endogenous depression without psychotic symptoms |
| E11y000 | 11596 | Unspecified manic-depressive psychoses |
| Eu32000 | 11717 | [X]Mild depressive episode |
| Eu41200 | 11913 | [X]Mixed anxiety and depressive disorder |
| Eu32300 | 12099 | [X]Severe depressive episode with psychotic symptoms |
| E115.11 | 12831 | Manic-depressive - now depressed |
| Eu53011 | 13307 | [X]Postnatal depression NOS |
| E113200 | 14709 | Recurrent major depressive episodes, moderate |
| E113.00 | 15099 | Recurrent major depressive episode |
| E112200 | 15155 | Single major depressive episode, moderate |
| E112300 | 15219 | Single major depressive episode, severe, without psychosis |
| Eu34114 | 15220 | [X]Persistant anxiety depression |
| E115000 | 15923 | Bipolar affective disorder, currently depressed, unspecified |
| E112100 | 16506 | Single major depressive episode, mild |
| Eu31300 | 16562 | [X]Bipolar affect disorder cur epi mild or moderate depressn |
| E291.00 | 16632 | Prolonged depressive reaction |
| Eu33315 | 16861 | [X]Recurrent severe episodes of psychotic depression |
| E130.11 | 17770 | Psychotic reactive depression |
| Eu32.12 | 18510 | [X]Single episode of psychogenic depression |
| Eu3y111 | 19054 | [X]Recurrent brief depressive episodes |
| Eu33.12 | 19696 | [X]Recurrent episodes of psychogenic depression |
| Eu20400 | 20785 | [X]Post-schizophrenic depression |
| E002100 | 21887 | Senile dementia with depression |
| ZV11112 | 22080 | [V]Personal history of manic-depressive psychosis |
| Eu33400 | 22116 | [X]Recurrent depressive disorder, currently in remission |
| Eu32212 | 22806 | [X]Single episode major depression w'out psychotic symptoms |
| Eu31400 | 23713 | [X]Bipol aff disord, curr epis sev depress, no psychot symp |
| Eu33311 | 23731 | [X]Endogenous depression with psychotic symptoms |
| ZV11111 | 23963 | [V]Personal history of manic-depressive psychosis |
| Eu32313 | 24112 | [X]Single episode of psychotic depression |
| Eu32311 | 24117 | [X]Single episode of major depression and psychotic symptoms |
| E113400 | 24171 | Recurrent major depressive episodes, severe, with psychosis |
| 1BQ..00 | 25435 | Loss of capacity for enjoyment |
| E113z00 | 25563 | Recurrent major depressive episode NOS |
| E113300 | 25697 | Recurrent major depressive episodes, severe, no psychosis |
| 1BT..12 | 26028 | Sad mood |
| E11y200 | 27491 | Atypical depressive disorder |
| E001300 | 27677 | Presenile dementia with depression |
| Eu02z16 | 27759 | [X] Senile dementia, depressed or paranoid type |
| E115200 | 27890 | Bipolar affective disorder, currently depressed, moderate |
| Eu32z13 | 28248 | [X]Prolonged single episode of reactive depression |
| Eu33312 | 28677 | [X]Manic-depress psychosis,depressed type+psychotic symptoms |
| Eu33.14 | 28756 | [X]Seasonal depressive disorder |
| Eu32314 | 28863 | [X]Single episode of reactive depressive psychosis |
| E113100 | 29342 | Recurrent major depressive episodes, mild |
| Eu33213 | 29451 | [X]Manic-depress psychosis,depressd,no psychotic symptoms |
| Eu33100 | 29520 | [X]Recurrent depressive disorder, current episode moderate |
| R007z13 | 29527 | [D]Postoperative depression |
| Eu33000 | 29784 | [X]Recurrent depressive disorder, current episode mild |
| Eu3y011 | 30688 | [X]Mixed affective episode |
| 1BP..00 | 30740 | Loss of interest |
| Eu33314 | 31757 | [X]Recurr severe episodes/psychogenic depressive psychosis |
| E112400 | 32159 | Single major depressive episode, severe, with psychosis |
| Eu33313 | 32941 | [X]Recurr severe episodes/major depression+psychotic symptom |
| Eu33200 | 33469 | [X]Recurr depress disorder cur epi severe without psyc sympt |
| Eu31z00 | 33751 | [X]Bipolar affective disorder, unspecified |
| E112000 | 34390 | Single major depressive episode, unspecified |
| E115300 | 35607 | Bipolar affect disord, now depressed, severe, no psychosis |
| E113000 | 35671 | Recurrent major depressive episodes, unspecified |
| E115100 | 35734 | Bipolar affective disorder, currently depressed, mild |
| E290z00 | 36246 | Brief depressive reaction NOS |
| Eu33z11 | 36616 | [X]Monopolar depression NOS |
| E115z00 | 37296 | Bipolar affective disorder, currently depressed, NOS |
| Eu33316 | 37764 | [X]Recurrent severe episodes/reactive depressive psychosis |
| E002z00 | 41089 | Senile dementia with depressive or paranoid features NOS |
| Eu32211 | 41989 | [X]Single episode agitated depressn w'out psychotic symptoms |
| E004300 | 43292 | Arteriosclerotic dementia with depression |
| E112500 | 43324 | Single major depressive episode, partial or unspec remission |
| Eu33z00 | 44300 | [X]Recurrent depressive disorder, unspecified |
| E002.00 | 44674 | Senile dementia with depressive or paranoid features |
| Eu31600 | 44693 | [X]Bipolar affective disorder, current episode mixed |
| Eu33300 | 47009 | [X]Recurrent depress disorder cur epi severe with psyc symp |
| Eu33y00 | 47731 | [X]Other recurrent depressive disorders |
| Eu32312 | 52678 | [X]Single episode of psychogenic depressive psychosis |
| 1BU..00 | 53148 | Loss of hope for the future |
| Eu31y00 | 53840 | [X]Other bipolar affective disorders |
| E113600 | 55384 | Recurrent major depressive episodes, in full remission |
| E113500 | 56273 | Recurrent major depressive episodes,partial/unspec remission |
| Eu32y12 | 56609 | [X]Single episode of masked depression NOS |
| E115600 | 57465 | Bipolar affective disorder, now depressed, in full remission |
| Eu32213 | 59386 | [X]Single episode vital depression w'out psychotic symptoms |
| 1BP0.00 | 59869 | Loss of interest in previously enjoyable activity |
| E11y.00 | 60178 | Other and unspecified manic-depressive psychoses |
| E115400 | 63701 | Bipolar affect disord, now depressed, severe with psychosis |
| E115500 | 72026 | Bipolar affect disord, now depressed, part/unspec remission |
| Eu31y11 | 73924 | [X]Bipolar II disorder |
| Eu33214 | 73991 | [X]Vital depression, recurrent without psychotic symptoms |
| Eu32600 | 98252 | [X]Major depression, moderately severe |
| Eu32500 | 98346 | [X]Major depression, mild |
| Eu32700 | 98414 | [X]Major depression, severe without psychotic symptoms |
| Eu32800 | 98417 | [X]Major depression, severe with psychotic symptoms |
| Anxiety: | | |
| 1B13.00 | 131 | Anxiousness |
| E200111 | 462 | Panic attack |
| 1B12.12 | 514 | Tension - nervous |
| E200.00 | 636 | Anxiety states |
| E200300 | 655 | Anxiety with depression |
| E20z.11 | 791 | Nervous breakdown |
| Eu41111 | 962 | [X]Anxiety neurosis |
| E205.11 | 1582 | Nervous exhaustion |
| E200400 | 1758 | Chronic anxiety |
| R2y2.00 | 2509 | [D]Nervousness |
| 1BK..00 | 2524 | Worried |
| E202100 | 3076 | Agoraphobia with panic attacks |
| 1B1..00 | 3328 | General nervous symptoms |
| E200100 | 4069 | Panic disorder |
| Eu41012 | 4081 | [X]Panic state |
| E200z00 | 4534 | Anxiety state NOS |
| E200500 | 4634 | Recurrent anxiety |
| E200200 | 4659 | Generalised anxiety disorder |
| Eu41.00 | 5385 | [X]Other anxiety disorders |
| 1B13.11 | 5902 | Anxiousness - symptom |
| E292000 | 6221 | Separation anxiety disorder |
| Eu41011 | 6408 | [X]Panic attack |
| E200000 | 6939 | Anxiety state unspecified |
| Eu41211 | 7749 | [X]Mild anxiety depression |
| Z4L1.00 | 7999 | Anxiety counselling |
| Eu41000 | 8205 | [X]Panic disorder [episodic paroxysmal anxiety] |
| Eu60600 | 8424 | [X]Anxious [avoidant] personality disorder |
| 2259.00 | 8725 | O/E - nervous |
| Eu41100 | 10344 | [X]Generalized anxiety disorder |
| E202D00 | 10390 | Fear of death |
| R2y2.12 | 10723 | [D]Nervous tension |
| 1B1V.00 | 11890 | C/O - panic attack |
| Eu41200 | 11913 | [X]Mixed anxiety and depressive disorder |
| E280.00 | 11940 | Acute panic state due to acute stress reaction |
| E202200 | 12838 | Agoraphobia without mention of panic attacks |
| 2258.00 | 13124 | O/E - anxious |
| Eu40012 | 14890 | [X]Panic disorder with agoraphobia |
| Eu40011 | 16729 | [X]Agoraphobia without history of panic disorder |
| Eu51511 | 17687 | [X]Dream anxiety disorder |
| 225J.00 | 19000 | O/E - panic attack |
| 1B1Z.00 | 20089 | General nervous symptom NOS |
| 1B1H.12 | 20163 | Apprehension |
| Eu41z00 | 23838 | [X]Anxiety disorder, unspecified |
| Eu41y00 | 24066 | [X]Other specified anxiety disorders |
| Eu41z11 | 25638 | [X]Anxiety NOS |
| 225K.00 | 26331 | O/E - fearful mood |
| Eu41y11 | 28167 | [X]Anxiety hysteria |
| Z4I7200 | 28381 | Alleviating anxiety |
| 8HHp.00 | 28925 | Referral for guided self-help for anxiety |
| 1B12.00 | 29608 | 'Nerves' - nervousness |
| Eu40z00 | 34064 | [X]Phobic anxiety disorder, unspecified |
| Eu41112 | 35825 | [X]Anxiety reaction |
| 2255.00 | 38155 | O/E - afraid |
| 1B1P000 | 40431 | Cries easily |
| Eu41300 | 44321 | [X]Other mixed anxiety disorders |
| Eu41113 | 50191 | [X]Anxiety state |
| E292400 | 56924 | Adjustment reaction with anxious mood |
| 1B13.12 | 93401 | Anxious |
| 16ZB100 | 101422 | Feeling low or worried |
| Neuropathic pain: | | |
| F262500 | 321 | Periodic migrainous neuralgia |
| F301.00 | 1541 | Other specified trigeminal neuralgia |
| A531.11 | 1598 | Post-herpetic neuralgia |
| N242000 | 2284 | Neuralgia unspecified |
| F301z00 | 6581 | Trigeminal neuralgia NOS |
| F356100 | 6884 | Morton's neuralgia |
| F300.00 | 7584 | Post-herpetic trigeminal neuralgia |
| A531511 | 10223 | Postherpetic neuralgia |
| A531200 | 11498 | Postherpetic trigeminal neuralgia |
| N242300 | 11544 | Neuropathic pain |
| 1475.00 | 16481 | H/O: trigeminal neuralgia |
| F321.00 | 16932 | Glossopharyngeal neuralgia |
| A531500 | 17180 | Postzoster neuralgia |
| N242z00 | 23839 | Neuralgia, neuritis or radiculitis NOS |
| F262100 | 33362 | Horton's (histamine) neuralgia |
| F372100 | 35785 | Chronic painful diabetic neuropathy |
| F372000 | 48078 | Acute painful diabetic neuropathy |
| N242.00 | 54992 | Neuralgia, neuritis and radiculitis unspecified |

*There is a one-to-one correspondence between Medcode and Read code in Clinical Practice Research Datalink.
