# Supplementary material for: Prevalence, incidence, indication, and choice of antidepressants in patients with and without chronic kidney disease: a matched cohort study in UK Clinical Practice Research Datalink
Source: Pharmacoepidemiol Drug Saf. 2017 Apr 11;26(7):792–801. doi: 10.1002/pds.4212 (PMC5516188; doi:10.1002/pds.4212)
Supplement: Supplementary file 2 — Appendix S2. Subgroup analyses according to level of kidney function (among patients with CKD) and creatinine measurement (among patients without CKD). [file PDS-26-792-s002.docx]

**Appendix 2. Subgroup analyses according to level of kidney function (among patients with CKD) and creatinine measurement (among patients without CKD).**

**Table 1**. Baseline characteristics.

|  | Patients without CKD (N = 242,349) | | Patients with CKD (N = 242,349) | | P value |
| --- | --- | --- | --- | --- | --- |
|  | without creatinine measurement in CPRD  N = 62,971 | with creatinine measurement in CPRD  N = 179,378 | with eGFR 30-59 mL/min/1.73m^2^ at baseline  N = 228,055 | with eGFR <30  mL/min/1.73m^2^ at baseline  N = 14,294 |  |
|  | n (%) | n (%) | n (%) | n (%) |  |
| Age (years): |  |  |  |  | <0.001 |
| <55 | 3,279 (5.2) | 3,566 (2.0) | 6,022 (2.6) | 823 (5.8) |  |
| 55-64 | 7,693 (12.2) | 15,863 (8.8) | 22,531 (9.9) | 1,025 (7.2) |  |
| 65-74 | 17,450 (27.7) | 53,662 (29.9) | 68,494 (30.0) | 2,618 (18.3) |  |
| 75-84 | 23,536 (37.4) | 79,058 (44.1) | 96,868 (42.5) | 5,726 (40.1) |  |
| ≥85 | 11,013 (17.5) | 27,229 (15.2) | 34,140 (15.0) | 4,102 (28.7) |  |
| Sex (male): | 23,015 (36.6) | 72,303 (40.3) | 89,289 (39.2) | 6,029 (42.2) | <0.001 |
| Ethnicity: |  |  |  |  | <0.001 |
| White/not-recorded | 62,319 (99.0) | 176,214 (98.2) | 224,211 (98.3) | 13,927 (97.3) |  |
| South Asian | 302 (0.5) | 1,494 (0.8) | 2,141 (0.9) | 176 (1.2) |  |
| Black | 146 (0.2) | 1,010 (0.6) | 932 (0.4) | 128 (0.9) |  |
| Other ethnicity | 204 (0.3) | 660 (0.4) | 771 (0.3) | 63 (0.4) |  |
| Socio-economic status: |  |  |  |  | <0.001 |
| 1 (least deprived) | 14,724 (23.4) | 42,076 (23.5) | 50,295 (22.1) | 2,739 (19.2) |  |
| 2 | 15,603 (24.8) | 46,044 (25.7) | 57,190 (25.1) | 3,311 (23.2) |  |
| 3 | 12,950 (20.6) | 37,516 (20.9) | 47,616 (20.9) | 3,093 (21.6) |  |
| 4 | 11,222 (17.8) | 30,999 (17.3) | 41,829 (18.3) | 2,863 (20.0) |  |
| 5 (most deprived) | 8,472 (13.5) | 22,743 (12.7) | 31,125 (13.7) | 2,288 (16.0) |  |
| Smoking status: |  |  |  |  | <0.001 |
| Non-smoker | 27,736 (44.1) | 64,627 (36.0) | 75,701 (33.2) | 5,020 (35.1) |  |
| Ex-smoker | 18,549 (29.5) | 89,188 (49.7) | 124,290 (54.5) | 7,220 (50.5) |  |
| Current-smoker | 11,791 (18.7) | 24,547 (13.7) | 27,374 (12.0) | 1,869 (13.1) |  |
| Missing | 4,895 (7.8) | 1,016 (0.6) | 690 (0.3) | 185 (1.3) |  |
| Body mass index: |  |  |  |  | <0.001 |
| <18.5 | 1,628 (2.6) | 5,010 (2.8) | 4,189 (1.8) | 373 (2.6) |  |
| 18.5 - 25 | 21,981 (34.9) | 63,492 (35.4) | 65,841 (28.9) | 4,261 (29.8) |  |
| ≥25 | 17,526 (27.8) | 62,932 (35.1) | 83,733 (36.7) | 4,350 (30.4) |  |
| ≥30 | 6,829 (10.8) | 33,497 (18.7) | 59,910 (26.3) | 3,273 (22.9) |  |
| Missing | 15,007 (23.8) | 14,447 (8.1) | 14,382 (6.3) | 2,037 (14.3) |  |
| Chronic physical illnesses: |  |  |  |  |  |
| Diabetes mellitus | 669 (1.1) | 23,623 (13.2) | 49,017 (21.5) | 3,785 (26.5) | <0.001 |
| Congestive heart failure | 824 (1.3) | 6,757 (3.8) | 20,723 (9.1) | 3,051 (21.3) | <0.001 |
| Myocardial infarction | 783 (1.2) | 10,676 (6.0) | 23,664 (10.4) | 2,082 (14.6) | <0.001 |
| Stroke | 1,507 (2.4) | 10,736 (6.0) | 18,330 (8.0) | 1,652 (11.6) | <0.001 |
| Chronic obstructive pulmonary disease | 2,312 (3.7) | 12,684 (7.1) | 17,006 (7.5) | 1,223 (8.6) | <0.001 |
| Rheumatoid arthritis | 527 (0.8) | 3,743 (2.1) | 5,674 (2.5) | 357 (2.5) | <0.001 |
| Cancer | 8,593 (13.7) | 38,838 (21.7) | 50,799 (22.3) | 3,651 (25.5) | <0.001 |
| Parkinson’s disease | 500 (0.8) | 2,191 (1.2) | 2,143 (0.9) | 150 (1.1) | <0.001 |
| Epilepsy | 670 (1.1) | 3,302 (1.8) | 3,450 (1.5) | 232 (1.6) | <0.001 |
| CKD = chronic kidney function, eGFR = estimated glomerular filtration rate. | | | | | |

**Table 2**. Prevalence of antidepressant prescription.

|  | No. of patients receiving antidepressants in the past 6 months | Prevalence,  % (95% CI) | Adjusted odds ratio (95% CI) | | |
| --- | --- | --- | --- | --- | --- |
|  |  |  | Model 1* | Model 2** | Model 3*** |
| Non-CKD patients without creatinine measurement in CPRD (N = 62,971) | 4,515 | 7.2 (7.0 – 7.4) | 0.49 (0.47 – 0.51) | 0.48 (0.46 – 0.50) | 0.52 (0.49 – 0.54) |
| Non-CKD patients with creatinine measurement in CPRD (N = 179,378) | 24,223 | 13.5 (13.3 – 13.7) | 1 (Reference) | 1 (Reference) | 1 (Reference) |
| CKD patients with eGFR 30-59 mL/min/1.73m^2^ at baseline (N = 228,055) | 36,815 | 16.1 (16.0 – 16.3) | 1.24 (1.22 – 1.27) | 1.23 (1.21 – 1.26) | 1.19 (1.16 – 1.21) |
| CKD patients with eGFR <30 mL/min/1.73m^2^ at baseline (N = 14,294) | 2,613 | 18.3 (17.6 – 18.9) | 1.35 (1.26 – 1.44) | 1.31 (1.23 – 1.41) | 1.20 (1.12 – 1.29) |
| CI = confidence interval, CKD = chronic kidney disease, eGFR = estimated glomerular filtration rate.  *Model 1: Adjusted by age, sex and financial year, and taking account of clustering by general practices with robust standard errors using unconditional logistic regression analysis.  **Model 2: Model 1 + adjusted by ethnicity, socio-economic status, smoking status and body mass index.  ***Model 3: Model 2 + adjusted by chronic physical illnesses. | | | | | |

**Table 3**. Incidence of new antidepressant prescription.

|  | Total follow-up length  (person-years) | No. of patients  starting antidepressants | Incidence rate  (/1000 person-years)  (95%CI) | Adjusted rate ratio (95%CI) | | |
| --- | --- | --- | --- | --- | --- | --- |
|  |  |  |  | Model 1** | Model 2*** | Model 3*** |
| Non-CKD patients without creatinine measurement in CPRD (N = 58,456) | 258,474 | 7,076 | 27.4 (26.7 – 28.0) | 0.55 (0.53 – 0.56) | 0.58 (0.56 – 0.59) | 0.60 (0.59 – 0.62) |
| Non-CKD patients with creatinine measurement in CPRD (N = 155,155) | 516,186 | 25,770 | 49.9 (49.3 – 50.5) | 1 (Reference) | 1 (Reference) | 1 (Reference) |
| CKD patients with eGFR 30-59 mL/min/1.73m^2^ (N = 191,240) | 762,310 | 43,410 | 56.9 (56.4 – 57.5) | 1.14 (1.12 – 1.16) | 1.13 (1.11 – 1.15) | 1.10 (1.09 – 1.12) |
| CKD patients with eGFR <30 mL/min/1.73m^2^ (N = 11,681) | 31,839 | 1,984 | 62.3 (59.6 – 65.1) | 1.24 (1.18 – 1.30) | 1.23 (1.17 – 1.28) | 1.16 (1.11 – 1.22) |
| CI = confidence interval, CKD = chronic kidney disease, eGFR = estimated glomerular filtration rate, IQR = interquartile range.  *Model 1: Adjusted by age, sex and financial year, and taking account of clustering by general practices with robust standard errors using unconditional Poisson regression analysis.  **Model 2: Model 1 + adjusted by ethnicity, socio-economic status, smoking status and body mass index.  ***Model 3: Model 2 + adjusted by chronic physical illnesses. | | | | | | |

**Table 4**. Recorded diagnoses for patients prescribed antidepressants stratified by type of antidepressant.

|  | Patients without CKD (N = 32,846) | | | | | |
| --- | --- | --- | --- | --- | --- | --- |
|  | without creatinine measurement in CPRD  (N = 7,076) | | | with creatinine measurement in CPRD  (N = 25,770) | | |
|  | SSRI  N = 2,984 | TCA  N = 3,591 | Others  N = 501 | SSRI  N = 9,940 | TCA  N = 14,081 | Others  N = 1,749 |
| Depression, n (%)* | 1,741 (58.3) | 759 (21.1) | 197 (39.3) | 6,382 (64.2) | 3,671 (26.1) | 838 (47.9) |
| Anxiety, n (%)* | 1,030 (34.5) | 646 (18.0) | 133 (26.6) | 3,813 (38.4) | 3,256 (23.1) | 575 (32.9) |
| Neuropathic pain, n (%)* | 76 (2.6) | 478 (13.3) | 19 (3.8) | 549 (5.5) | 2,058 (14.6) | 133 (7.6) |
| None of the above, n (%) | 850 (28.5) | 2,149 (59.8) | 244 (48.7) | 2,338 (23.5) | 7,550 (53.6) | 650 (37.2) |

|  | Patients with CKD (N = 45,394) | | | | | |
| --- | --- | --- | --- | --- | --- | --- |
|  | eGFR 30-59 mL/min/1.73m^2^  (N = 43,410) | | | eGFR <30 mL/min/1.73m^2^  (N = 1,984) | | |
|  | SSRI  N = 17,124 | TCA  N = 23,286 | Others  N = 3,000 | SSRI  N = 868 | TCA  N = 976 | Others  N = 140 |
| Depression, n (%)* | 10,871 (63.5) | 6,017 (25.8) | 1,390 (46.3) | 492 (56.7) | 240 (24.6) | 66 (47.1) |
| Anxiety, n (%)* | 5,904 (34.5) | 4,874 (20.9) | 897 (29.9) | 227 (26.2) | 181 (18.6) | 38 (27.1) |
| Neuropathic pain, n (%)* | 942 (5.5) | 3,348 (14.4) | 201 (6.7) | 55 (6.3) | 143 (14.7) | 8 (5.7) |
| None of the above, n (%) | 4,395 (25.7) | 12,715 (54.6) | 1,195 (39.8) | 288 (33.2) | 544 (55.7) | 61 (43.6) |
| CKD = chronic kidney disease, eGFR = estimated glomerular filtration rate, SSRI = selective serotonin reuptake inhibitor, TCA = tricyclic antidepressants.  *Percentages are column percentages. Each patient may have one or more recorded diagnosis. | | | | | | |

**Table 5**. Choice of antidepressants and initial prescription dose for patients with diagnosed depression.

|  | Patients without CKD (N = 13,588) | | | | Patients with CKD (N = 19,076) | | | |
| --- | --- | --- | --- | --- | --- | --- | --- | --- |
|  | without creatinine measurement  in CPRD  N = 2,697 | | with creatinine measurement  in CPRD  N = 10,891 | | with eGFR 30-59  mL/min/1.73m^2^ at baseline  N = 18,278 | | with eGFR <30  mL/min/1.73m^2^ at baseline  N = 798 | |
|  | n (%)* | Median initial dose (mg/day) [IQR] | n (%)* | Median initial dose (mg/day) [IQR] | n (%)* | Median initial dose (mg/day) [IQR] | n (%)* | Median initial dose (mg/day) [IQR] |
| Selective serotonin reuptake inhibitors |  |  |  |  |  |  |  |  |
| Citalopram | 1,051 (39.0) | 10 [10 – 20] | 3,883 (35.7) | 10 [10 – 20] | 6,760 (37.0) | 10 [10 – 20] | 310 (38.9) | 10 [10 – 20] |
| Escitalopram | 80 (3.0) | 10 [5 – 10] | 273 (2.5) | 5 [5 – 10] | 407 (2.2) | 5 [5 – 10] | 22 (2.8) | 5 [5 – 10] |
| Fluoxetine | 381 (14.3) | 20 [20 – 20] | 1,270 (11.7) | 20 [20 – 20] | 2,171 (11.9) | 20 [20 – 20] | 99 (12.4) | 20 [20 – 20] |
| Fluvoxamine | <5 (<0.2) | n/a | <5 (<0.1) | n/a | <5 (<0.1) | n/a | <5 (<0.6) | n/a |
| Paroxetine | 35 (1.3) | 20 [20 – 20] | 97 (0.9) | 20 [20 – 20] | 133 (0.7) | 20 [20 – 20] | 11 (1.4) | 20 [20 – 20] |
| Sertraline | 194 (7.2) | 50 [50 – 50] | 859 (7.9) | 50 [50 – 50] | 1,399 (7.7) | 50 [50 – 50] | 50 (6.3) | 50 [50 – 50] |
| Tricyclic and related antidepressants |  |  |  |  |  |  |  |  |
| Amitriptyline | 548 (20.3) | 10 [10 – 15] | 2,958 (27.2) | 10 [10 – 20] | 4,847 (26.5) | 10 [10 – 15] | 177 (22.2) | 10 [10 – 15] |
| Clomipramine | <5 (<0.2) | n/a | 22 (0.2) | 20 [10 – 37.5] | 27 (0.2) | 20 [10 – 37.5] | <5 (<0.6) | n/a |
| Dosulepin | 105 (3.9) | 50 [25 – 75] | 302 (2.8) | 37.5 [25 – 75] | 481 (2.6) | 37.5 [25 – 50] | 31 (3.9) | 37.5 [25 – 75] |
| Doxepin | <5 (<0.2) | n/a | 17 (0.2) | 25 [25 – 37.5] | 23 (0.1) | 25 [20 – 25] | <5 (<0.6) | n/a |
| Imipramine | <5 (<0.2) | n/a | 27 (0.3) | 15 [10 – 25] | 44 (0.2) | 25 [10 – 30] | <5 (<0.6) | n/a |
| Lofepramine | 26 (1.0) | 70 [70 – 140] | 87 (0.8) | 70 [70 – 140] | 179 (1.0) | 70 [70 – 140] | 7 (0.9) | 70 [70 – 140] |
| Nortriptyline | 10 (0.4) | 15 [10 – 25] | 84 (0.8) | 15 [10 – 15] | 155 (0.9) | 10 [10 – 15] | <5 (<0.6) | n/a |
| Trimipramine | <5 (<0.2) | n/a | 12 (0.1) | 25 [15 – 37.5] | 24 (0.1) | 30 [15 – 50] | <5 (<0.6) | n/a |
| Mianserin | <5 (<0.2) | n/a | <5 (<0.1) | n/a | <5 (<0.1) | n/a | <5 (<0.6) | n/a |
| Trazodone | 55 (2.0) | 50 [50 – 100] | 158 (1.5) | 50 [50 – 100] | 233 (1.3) | 50 [50 – 75] | 17 (2.1) | 50 [50 – 75] |
| Monoamine oxidase inhibitors** | <5 (<0.2) | n/a | <5 (<0.1) | n/a | <5 (<0.1) | n/a | <5 (<0.6) | n/a |
| Other antidepressants: |  |  |  |  |  |  |  |  |
| Agomelatine | <5 (<0.2) | n/a | <5 (<0.1) | n/a | <5 (<0.1) | n/a | <5 (<0.6) | n/a |
| Duloxetine | 15 (0.6) | 60 [40 – 60] | 83 (0.8) | 40 [30 – 60] | 164 (0.9) | 40 [30 – 60] | 5 (0.6) | 60 [60 – 60] |
| Flupentixol | 17 (0.6) | 1 [0.5 – 1] | 46 (0.4) | 0.5 [0.5 – 1] | 82 (0.5) | 1 [0.5 – 1] | 6 (0.8) | 0.5 [0.5 – 0.5] |
| Mirtazapine | 139 (5.2) | 15 [15 – 15] | 619 (5.7) | 15 [15 – 15] | 998 (5.5) | 15 [15 – 15] | 47 (5.9) | 15 [15 – 15] |
| Reboxetine | <5 (<0.2) | n/a | <5 (<0.1) | n/a | <5 (<0.1) | n/a | <5 (<0.6) | n/a |
| Venlafaxine | 19 (0.7) | 75 [75 – 75] | 66 (0.6) | 75 [75 – 75] | 94 (0.5) | 75 [75 – 75] | <5 (<0.6) | n/a |
| Two or more different antidepressants | 7 (0.3) | n/a | 20 (0.2) | n/a | 48 (0.3) | n/a | 5 (0.6) | n/a |
| CKD = chronic kidney disease, eGFR = estimated glomerular filtration rate, IQR = interquartile range.  *Cell counts less than five have been suppressed to preserve patient privacy.  **Phenelzine, isocarboxazid, tranylcypromine and moclobemide are combined due to small sample sizes. | | | | | | | | |
